# A simple method of spinal length assessment in patients with idiopathic scoliosis

**DOI:** 10.1186/1748-7161-8-S1-O8

**Published:** 2013-06-03

**Authors:** M Tyrakowski, D Wojtera-Tyrakowska, T Kotwicki, J Czubak

**Affiliations:** 1 Department of Orthopaedics, Pediatric Orthopaedics and Traumatology of the Medical Centre of Postgraduate Education, Warsaw, Poland; 2 Department of Mathematics and Computer Science, Adam Mickiewicz University, Poznan, Poland; 3 Spine Disorders Unit, Department of Pediatric Orthopedics and Traumatology, University of Medical Sciences, Poznan, Poland

## Background

The height of patients with idiopathic scoliosis (IS) is diminished due to the curvature of the spine. Several clinical parameters (BMI, vital capacity, others) are dependent on the patient’s height [1,2]. We developed a formula to calculate the corrected length of the spine in patients with IS, based on the presumption that scoliotic curve may be considered a part of a circle.

## Aim

The aim of the study was to calculate the corrected length of the spine using our own formula, and compare it with the length directly measured on radiographs.

## Methods

On the AP long film standing radiographs of 40 consecutive patients, undergoing surgery for IS, the Cobb angle (α) and the direct distance (h) between the upper end vertebra and the lower end vertebra (centroid of vertebral body) were measured. The length of the spinal curvature (c) was calculated in the computer program using the formula: c=αh/2sin^α^/_2_ For each patient the calculated length was compared to the length of the curvature measured on the radiograph. Shapiro-Wilk W test, t-Student’s test, and Pearson’s linear correlation were used.

## Results

There was no statistically significant difference between the length of the scoliotic curve measured on radiograph and the length calculated with software (p=0.54). A strong correlation between these two parameters was found, Pearson linear correlation coefficient 0.98.

## Conclusions

The method of correcting the spine length according to the Cobb angle seems to be simple and accurate. It needs only one additional parameter measured on the radiograph – the distance between the two end vertebrae. However, it concerns only the frontal plane. The software may be used on personal computers as well as on mobile phones, and thus help in everyday clinical practice.

## References

[B1] Bjure J Grimby G Nachemson A Correction of body height in predicting spirometric values in scoliotic patients Scand J Clin Lab Invest 1968 21 2 191 192 5706641

[B2] Kono K Asazuma T Suzuki N Ono T Body height correction in scoliosis patients for pulmonary function test J Orthop Surg (Hong Kong) 2000 8 1 19 26 12468871 10.1177/230949900000800105

